# Case report of a squamous cell carcinoma arising in a vulvar cutaneous horn

**DOI:** 10.1016/j.gore.2025.101784

**Published:** 2025-06-18

**Authors:** Michael Bell, Erin Crane, Robert Tucker Burks

**Affiliations:** a Department of Obstetrics and Gynecology, Atrium Health Carolinas Medical Center, Charlotte, North Carolina, United States; b Wake Forest University School of Medicine, Department of Obstetrics and Gynecology, Department of Gynecologic Oncology, Atrium Health Carolinas Medical Center, Levine Cancer Institute, Charlotte, North Carolina, United States; c Carolinas Pathology Group, Charlotte, North Carolina, United States

**Keywords:** Cutaneous Horn, Vulvar cancer, Squamous Cell Carcinoma

## Abstract

**Background:**

Cutaneous horns are rare lesions that consist of a proliferation of keratotic material, often representing animal horns. Rarely, they may be associated with underlying squamous cell carcinomas. Here, we present a case report of a cutaneous horn arising in a squamous cell carcinoma of the vulva.

**Objective:**

In this report, we highlight the case of an 84 year-old patient who presented with a vulvar cutaneous horn. After resection, pathology demonstrated an invasive squamous cell carcinoma of the vulva. We provide a review of available literature and describe a rare manifestation of a squamous cell carcinoma of the vulva. Given her frailty, no further treatment was pursued, and she remains in observation without evidence of recurrence.

**Conclusions:**

Our case highlights a rare presentation of a vulvar cutaneous horn arising within invasive squamous cell carcinoma of the vulva. While most cutaneous horns are benign, a subset may harbor malignancy, and therefore excision is warranted. Additional treatment is dependent upon histopathology, disease site, and patient factors.

## Highlights

• Cutaneous horns are rare epithelial proliferations that bear resemblance to an animal horn.
• Cutaneous horns may harbor carcinomas and are treated with surgical resection.
• Here we describe an 84 year-old wo presented with a vulvar cutaneous horn that contained an invasive carcinoma.

## 1 Introduction

Cutaneous horns are rare lesions that consist of a proliferation of keratotic material which resembles an animal horn. A multitude of causes have been identified, ranging from benign to malignant disease, as well as reactive and infectious conditions. In the context of malignant or premalignant conditions, actinic keratoses in sun-exposed locations comprise the most common etiology (Mantese et al., 2010). These lesions may give rise to squamous cell carcinomas. As such, cutaneous horns are most commonly found on chronic sun damaged skin, particularly the scalp, face, ear, nose, forearm, and hands. Case reports also exist describing cutaneous horns on non sun-exposed areas (Pyne et al., 2013). Cutaneous horns occur most commonly between ages 60–80, and are more prevalent in males (Yu et al., 1991). The risk of underlying malignancy increases with age.

While most cutaneous horns arise on extremities and sun-exposed areas, there have been rare reports of penile cutaneous horns in the literature (Gupta et al., 2014, Pal and Pal, 2020). To date, there have been no case reports of cutaneous horns arising from the vulva. Here, we discuss a novel case of squamous cell carcinoma which manifested in a vulvar cutaneous horn and discuss management.

## 2 Case presentation

**Fig. 1:**
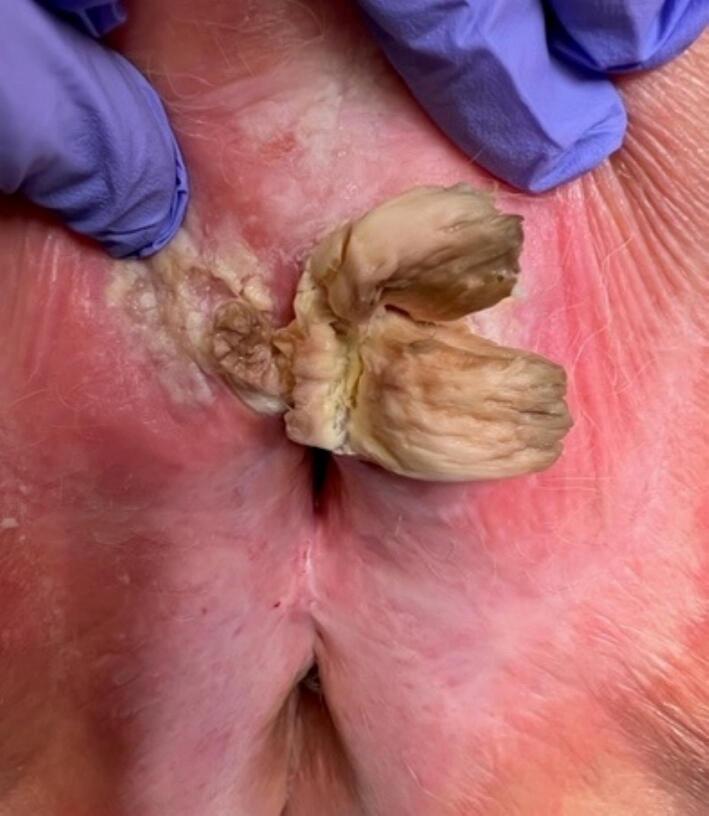
Exam prior to excision. Examination of the external genitalia, including labia majora and minora, anus, perineum, urethra and vaginal introitus revealed a 3 cm mass centered around the clitoris. It was cornified and raised, extending out 1 cm, with several distinct projections.

An 84-year-old G3P3 presented with a several-month history of vulvar irritation and a vulvar mass. Her gynecologist obtained a punch biopsy which initially showed squamous mucosa with hyperkeratosis, parakeratosis and milled to moderate epidermal and dermal chronic inflammatory infiltrate. The mass grew and over the ensuing months became more symptomatic, affecting urination. She was seen again by her gynecologist who appreciated a large mass and referred her to Gynecologic Oncology. Examination of the external genitalia revealed a 3 cm mass centered around the clitoris (Fig. 1). It was cornified and raised, extending out 1 cm, with several distinct projections. It appeared consistent with condyloma or verrucous carcinoma with surrounding erythema and lichenification. The remainder of the vulva appeared atrophic without lesions.

**Fig. 2:**
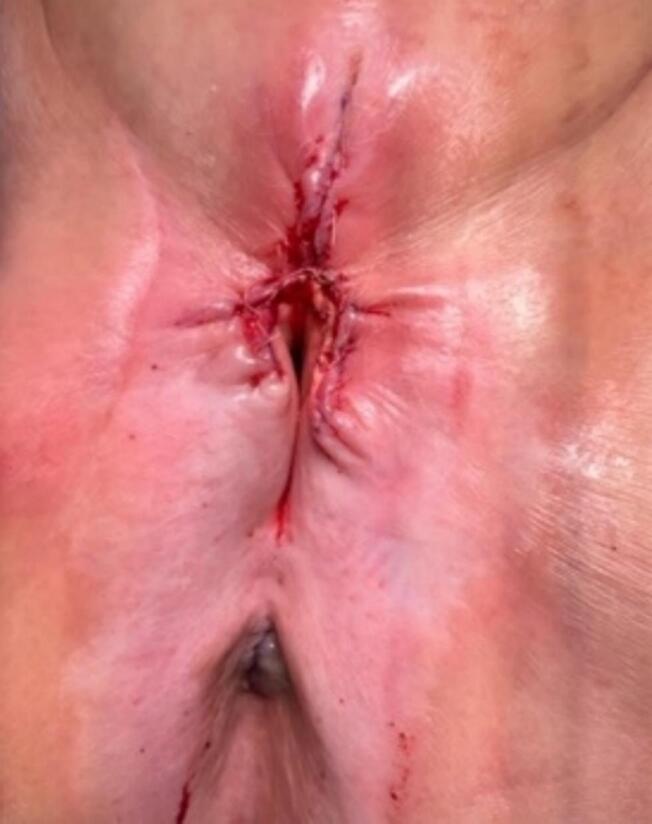
Postoperative result. Wide radical excision of the mass to include resection of the mass itself as well as a margin. Patient wastaken to OR and underwent radical partial vulvectomy. Intraoperatively, she had an exophytic cutaneous horn arising from the clitoris superior to the urethra, and a 10 x 4 cm area was resected. The lesion was sharply incised and bovie cautery was used to resect down to the level of the pubocervical fascia.

Based on the patient’s age and comorbidities including significant coronary artery disease, the decision was made to proceed with palliative excision rather than complete vulvectomy and/or lymph node assessment in the case of malignancy. The patient therefore underwent radical partial vulvectomy. Intraoperatively, there was an exophytic cutaneous horn arising from the clitoris superior to the urethra, and a 10 × 4 cm area was resected. The lesion was sharply incised and resected down to the level of the pubocervical fascia (Fig. 2).

Final pathology results revealed foci of invasive squamous cell carcinoma with greatest depth of invasion measuring up to 2 mm, arising in association with atypical verrucous squamoproliferative lesion arising in a background of lichen sclerosus (Fig. 3a, Fig. 3b). All margins were negative for invasive carcinoma, but the atypical verrucous squamous proliferation extended to 3 mm of the closest peripheral margin and focally abutted the deep margin. On the basis of clinical and histopathological findings, a diagnosis of stage IB squamous cell carcinoma of the vulva was made. Given the patient’s age and frailty, the decision was made to proceed with observation rather than return for re-excision or lymph node assessment. Imaging was offered to rule out metastatic disease but declined by the family. The patient was prescribed clobetasol proprionate for treatment of underlying lichen sclerosus and remains disease-free at the 1.5-year mark.

**Fig. 3a:**
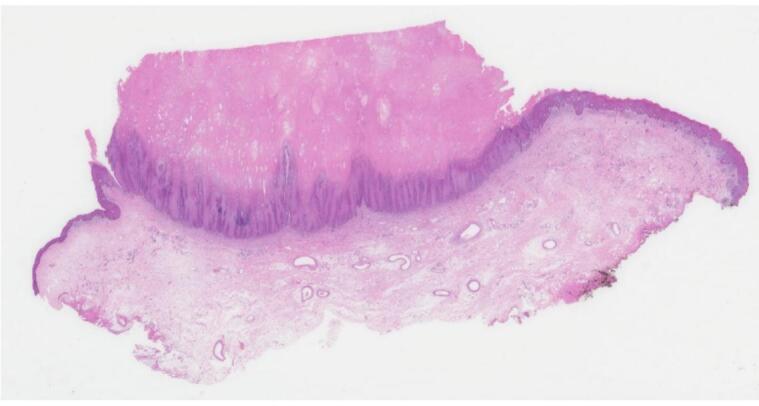
Cutaneous horn composed of lamellated keratotic material with underlying verruciform proliferation.

**Fig. 3b:**
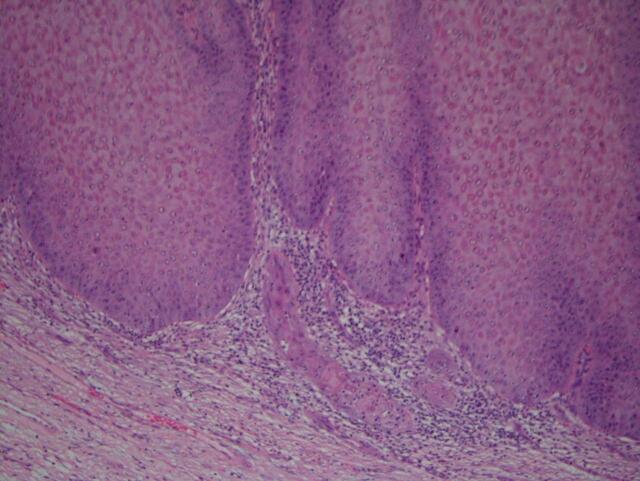
Minute focus of superficially invasive squamous cell carcinoma.

## 3 Discussion

This case highlights a rare presentation of a cutaneous horn with invasive squamous cell carcinoma of the vulva. To our knowledge, this is the first case report of a vulvar cutaneous horn affecting the female genitalia. While cutaneous horns have been described in association with human papilloma virus (Solivan et al., 1990), in this case the horn and underlying carcinoma were associated with lichen sclerosus, a condition which can predispose patients to vulvar squamous cell carcinomas (Vieira-Baptista et al., 2022). Chronic irritation may also contribute to malignant transformation of cutaneous horns (Karthikeyan et al., 1998), a process also described in malignant transformation of lichen sclerosus.

The earliest documentation of a cutaneous horn was in 1588, described in an older female as an “anomaly of nature”(Bondeson, 2001). Reports of cutaneous horns are rare, Yu et al provided the largest classification involving 643 cases. The majority (61.1 %) were benign, while 38.9 % harbored malignancy, most commonly squamous cell carcinoma but basal cell carcinomas were also described. Another review of 163 malignant cases identified risk factors associated with malignancy including horn height less than the diameter of the base, absence of terrace morphology, presence of erythema at the base of the horn, and the presence of pain (Pyne et al., 2013). This was consistent with the findings in our case, where the patient had pain, surrounding erythema, and a height of 1 cm with a wider (3 cm) base.

Management of cutaneous horns involves surgical excision, with careful examination of the base of the horn for an invasive malignancy. To date, there are no aggregate reports describing clinical outcomes of patients with squamous cell carcinomas arising from vulvar horns, but presumably clinical behavior is dictated by the malignant component rather than the horn itself. In this case, the patient met criteria for stage IB squamous cell carcinoma of the vulva. Standard of care for patients with stage IB disease includes radical partial vulvectomy and inguinofemoral lymph node evaluation. Based on the patient’s frailty and medical comorbidities, the decision was made to proceed with palliative resection alone. Imaging was offered to rule out metastatic disease but the patient’s family declined. Studies have associated tumor size and depth of invasion with the risk of lymph node metastases (Klapdor et al., 2019). In a large retrospective analysis of 1162 patients with vulvar cancer, tumor diameter of 1–2 mm was associated with a 14–20 % risk of inguinal lymph node metastases, thus highlighting the importance of lymph node assessment in patients with vulvar cancer who are appropriate for surgical management (Klapdor et al., 2019).

## 4 Conclusion

Here we present the first known reported case of a vulvar cutaneous horn giving rise to a squamous cell carcinoma. Cutaneous horns are managed with surgical excision, and if malignancy is discovered, subsequent management is dictated by histopathology.

## CRediT authorship contribution statement

**Michael Bell:** Writing – review & editing, Writing – original draft, Investigation, Data curation, Conceptualization. **Erin Crane:** Writing – review & editing, Supervision, Investigation. **Robert Tucker Burks:** Investigation, Formal analysis.

## Declaration of competing interest

The authors declare that they have no known competing financial interests or personal relationships that could have appeared to influence the work reported in this paper.
